# Muscone Ameliorates Ovariectomy-Induced Bone Loss and Receptor Activator of Nuclear Factor-κb Ligand-Induced Osteoclastogenesis by Suppressing TNF Receptor–Associated Factor 6-Mediated Signaling Pathways

**DOI:** 10.3389/fphar.2020.00348

**Published:** 2020-03-20

**Authors:** Xiao Zhai, Zijun Yan, Jian Zhao, Kai Chen, Yilin Yang, Mengxi Cai, Chen He, Chunyou Huang, Bo Li, Mingyuan Yang, Xiaoyi Zhou, Yingchuan Zhao, Xiaozhao Wei, Yushu Bai, Ming Li

**Affiliations:** ^1^Department of Orthopedics, Shanghai Changhai Hospital, Navy Medical University, Shanghai, China; ^2^Graduate Management Unit, Shanghai Changhai Hospital, Navy Medical University, Shanghai, China

**Keywords:** muscone, postmenopausal osteoporosis, osteoclasts, RANK, TRAF6

## Abstract

Postmenopausal osteoporosis is caused by the deficiency of estrogen, which breaks bone homeostasis and induces levels of pro-inflammatory cytokines. Muscone is a potent anti-inflammatory agent and is used to treat bone fracture in traditional Chinese medicine. However, its anti-osteoclastogenic effects remain unclear. For *in vitro* study, morphology tests of osteoclastogenesis were firstly performed. And then, factors in RANK-induced NF-κB and MAPK pathways were examined by RT-PCR and Western blot, and the binding of TNF receptor–associated factor (TRAF)6 to RANK was inspected by coimmunoprecipitation and immunofluorescence staining. For *in vivo* experiments, C57BL/6 ovariectomized (OVX) mice were used for detection, including H&E staining, TRAP staining, and micro CT. As a result, muscone reduced OVX-induced bone loss in mice and osteoclast differentiation *in vitro*, by inhibiting TRAF6 binding to RANK, and then suppressed NF-κB and MAPK signaling pathways. The expression of the downstream biomarkers was finally inhibited, including NFATc1, CTR, TRAP, cathepsin K, and MMP-9. The inflammatory factors, TNF-a and IL-6, were also reduced by muscone. Taken together, muscone inhibited the binding of TRAF6 to RANK induced by RANKL, thus blocking NF-kB and MAPK pathways, and down-regulating related gene expression. Finally, muscone inhibited osteoclastogenesis and osteoclast function by blocking RANK-TRAF6 binding, as well as downstream signaling pathways *in vitro*. Muscone also reduced ovariectomy-induced bone loss *in vivo*.

## Introduction

Postmenopausal osteoporosis (PMOP) has become a major public health burden in the aging society (Qiu et al., 2018), and strategies for the prevention and treatment are mainly focused on regulating bone homeostasis by increasing osteoblasts and suppressing osteoclasts (Gao et al., 2018; Mediero et al., 2018). After menopause, the deficiency of estrogen can elevate the levels of pro-inflammatory cytokines, and macrophage colony-stimulating factor (M-CSF) with receptor activator of nuclear factor-kB ligand (RANKL), and then increases the differentiation of osteoclasts which are derived from bone marrow monocytes (BMMs) (Chen et al., 2017; Chen et al., 2018). RANKL and its receptor RANK bind together, and subsequently recruit the TNF receptor-associated factors (TRAFs) to form the complex (RANK-TRAF6, in particular) (Cao, 2018). And then, multiple downstream signaling pathways are activated, including nuclear factor-kB (NF-kB), mitogen-activated protein kinase (MAPK), and AKT signaling pathways, causing released intracellular Ca2+ and activated nuclear factor of activated T-cell cytoplasmic 1 (NFATc1) (Grossinger et al., 2018). In the signaling network of osteoclastogenesis, NFATc1 is the essential transcriptional factor for downstream genetic transcriptions, including matrix metalloproteinase 9 (MMP-9), cathepsin K, calcitonin receptor (CTR), tartrate-resistant acid phosphatase (TRAP), and NFATc1 itself (Nakashima et al., 2011; Chen et al., 2018).

**Figure 1:**
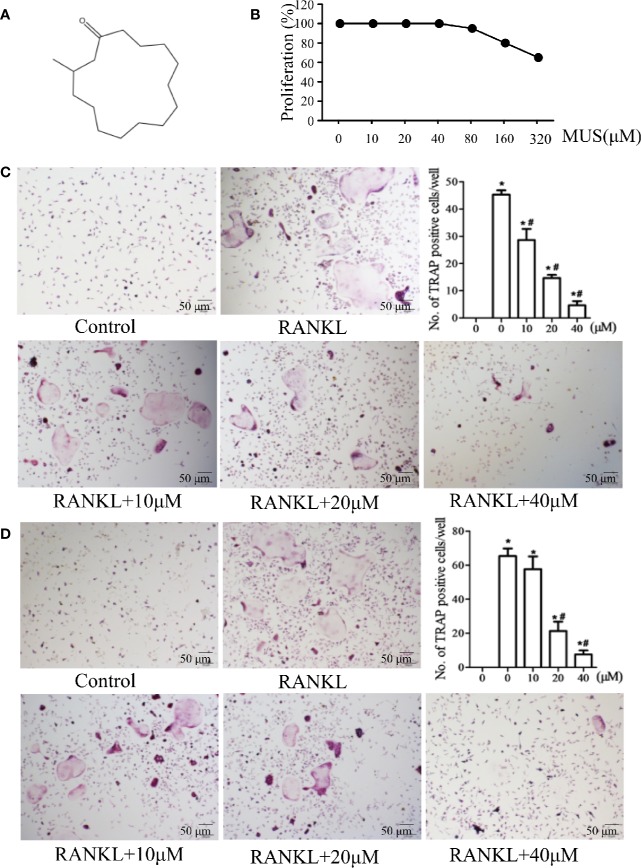
Muscone inhibits osteoclastogenesis in BMMs and the RAW264 cell line. **(A)** Chemical structure of muscone. **(B)** proliferation of muscone-exposed BMMs. **(C, D)** BMMs and RAW264.7 cells are used and treated with 10, 20, 40 μM muscone, and TRAP-positive cells are quantified (magnification ×100). *P < 0.05 vs. the control group, ^#^*P* < 0.05 vs. the RANKL-induced group.

To treat PMOP, we should reconstruct the bone homeostasis by increasing osteoblasts and suppressing osteoclasts. Several drugs such as vitamin D (Cianferotti et al., 2015) have been used, while each drug also has its own drawbacks. It is important to detect the new drugs, and many institutions are engaged into detecting the potential drugs (Santhosh et al., 2019). The natural pure compounds can be the potential drug candidates for the treatment and prevention of PMOP (Kushwaha et al., 2019; Yang et al., 2019). Muscone (MUS), 3-methylcyclopentadecanone, is the main ingredient of musk (**Figure 1A**). It is used to treat myocardial infarction (Wang et al., 2014; Du et al., 2018), relieve pain, and promote fractures (Guo et al., 2018). Furthermore, it has been proved to have anti-inflammatory effects on multiple systems (Wang et al., 2014; Liu et al., 2014). Given its promoting healing of the fractures and anti-inflammatory effects, muscone might prevent ovariectomy (OVX)-induced bone loss, and even serve as a potential alternative option on postmenopausal osteoporosis. Therefore, this study aimed to investigate the effects of muscone on osteoclast activity and explore the underlying molecular mechanisms.

## Methods

### Reagents and Antibodies

Muscone (purity > 98%) was (**Figure 1A**) provided by Yuanye Biotechnology (Shanghai, China). RAW264.7 and BMMs cells were provided by Professor Wang (Navy Medical University). Reagents and antibodies, such as penicillin-streptomycin, fetal bovine serum (FBS), RANK antibody, and p65 antibody, were purchased from Puhe Biotechnology (Wuxi, China).

### Cell Viability by MTT Assay

BMMs were cultured for 48 h with muscone at different concentrations (0, 10, 20, 40, 80, 160, and 320 μM). MTT solution was then added for 2 h. Finally, the absorbance was measured by enzyme-linked immunosorbent assay plate reader at 490 nm.

### Osteoclastogenesis Assay In Vitro

For primary cell culture, BMMs were isolated from the femur of C57BL/6 mice (Thummuri et al., 2017) and were treated with α-MEM and 30 ng/ml M-CSF for 3 days. The cells (1 × 10^4^ cells/well) were seeded into 96-well plates and induced by 20 ng/mL M-CSF and 50 ng/mL RANKL. Various concentrations of muscone (0, 10, 20, and 40 μM) were added into the plates on the day 0. After 7 days, tartrate-resistant acid phosphatase (TRAP) staining (Sigma, MO, USA) was performed, and TRAP-positive cells with more than 3 nuclei were counted as osteoclasts.

### Actin-Ring Formation Assay and Pit Formation Assay

The actin-ring formation assay was performed as described previously (Koide et al., 2009). Cells, induced by M-CSF and RANKL for 7 days, were fixed and washed three times by PBS. Treated with fluorescein isothiocyanate (FITC)-phalloidin for 1 h, cells were then stained.

The pit formation assay was done as previously described (Chen et al., 2017). The induced mature osteoclasts were processed by collagenase and seeded onto FBS-coated dentin slices in a 96-well plate with M-CSF and RANKL. Muscone was added into the plate. After 2 days, the slices were treated and stained with 0.5% toluidine blue for 1 min.

### Alkaline Phosphatase (ALP), Alizarin Red Staining (ARS), and Oil Red O Staining

With osteogenic differentiation medium, bone marrow-derived mesenchymal stem cells (BMSCs) were planted onto 24-well plates in the presence 0, 10, 20, and 40 μM MUS. Cells were washed 3 times with PBS, and were fixed with 4% paraformaldehyde for 15 min. It was 14 days and 21 days after the osteogenic induction that ALP staining (Sigma‐Aldrich) and ARS staining (Nanjing Jiancheng Chemical Industrial Co., Nanjing, China) were conducted, respectively.

Furthermore, to induce adipogenesis, BMSCs were cultured with 10% FBS α‐MEM supplied with 10 μg/mL insulin, 200 μmol/L indomethacin, 1 μmol/L dexamethasone, and 0.5 mmol/L 3‐isobutyl‐1‐methylxanthine (IBMX) (Cyagen Biosciences). Differentiated cells were then marked with Oil Red O staining (Sigma‐Aldrich).

Staining cells were observed under a microscope (Leica, Germany), and the mineralized area was analyzed by ImageJ software (National Institutes of Health, USA).

### Immunofluorescence Staining

The effects of muscone (40 μM) on the p65 nuclear translocation, RANK, and TRAF6 in RAW264.7 cells were examined by immunofluorescence described previously (Lee et al., 2010). Cells were fixed and washed. Antibodies of targeted factors were added with anti-mouse IgG antibody and fluorescein-conjugated streptavidin sequentially for further observing and imaging.

### Western Blotting and Coimmunoprecipitation

RAW264.7 cells were divided into 3 groups. All groups were treated with M-CSF, and two groups were treated with only RANKL or RANKL + MUS (40 μM). Related factors in RANK-induced NF-κB and MAPK pathways were examined as described previously (Chen et al., 2018).

To assess whether muscone influences the binding of RANK and TRAF6 induced by RANKL, coimmunoprecipitation experiments were performed with or without 40 μM muscone, as described previously (Hu et al., 2019). And then, Western blotting was performed and analyzed.

### Animal Model

Female C57BL/6 mice aged 8-week-old, weight 25 ± 1 g were obtained from Slack (Shanghai, China), and were maintained with free access to food and water. A total of 50 mice were divided into five groups (N=10 for each group) according to the method of random number table: control group, sham group, ovariectomy (OVX) group, muscone (MUS) treating group, and zoledronic acid (ZOL) treating group. The OVX model was performed in the specific pathogen free animal laboratory as described previously (Leng et al., 2005), in accordance with guidelines for reporting experiments involving animals. Muscone was injected intraperitoneally with 2 mg/kg every day and zoledronic acid (0.1mg/kg) (Hu et al., 2019) was injected subcutaneously once a week. 6 weeks later, mice were sacrificed by cervical dislocation, and then femurs were collected. And arterial blood samples were also reserved for further investigation. Ethical committee of Shanghai Changhai Hospital approved the treatment on animals.

### Histologic Analysis

H&E staining and TRAP staining were used for histologic analysis after femur bone samples were prepared and cut into 4-μm sections. Trabecular bone area and the number of osteoclasts were calculated by the image J software (National Institutes of Health, Bethesda, MD).

### Micro-Computed Tomography (micro-CT) Analysis

For each femur, 100 section planes from the growth plate were scanned (Skyscan1172, Antwerp, Belgium). We used built-in software to analyze index, including trabecular bone, bone mineral density (BMD), bone volume/total volume (BV/TV), bone surface area/total volume (BS/TV), and trabecular number (Tb.N), within the selected metaphyseal region. In addition, bone structure images in two-dimensional and three-dimensional version were reconstructed.

### Real-Time PCR

Cellular RNA was extracted using TRIzol (Invitrogen) as described previously (Li et al., 2011). PCR primers were shown in **Supplemental Table 1**.

### Statistical Analysis

All statistical analysis was performed using SAS 9.1 software. Results were repeated for five times and presented as means ± SEM. Results were compared with one way ANOVA followed by SNK test for multiple comparisons. *P* < 0.05 was regarded statistically significant.

## Results

### Muscone Inhibits Osteoclastogenesis in BMMs and the RAW264.7 Cell Line

Cell viability is examined by MTT assay. It shows that muscone has no obvious cytotoxic effects below 40 μM (**Figure 1B**). To examine the effects of muscone on osteoclastogenesis, BMMs and RAW264.7 cells are used and treated with 10, 20, and 40 μM muscone. As the results showed in **Figures 1C, D**, in the RANKL group, the number of TRAP-positive cells is significantly increased after RANKL induction within 5 to 7 days for BMMs and within 3 to 5 days for RAW264.7 cells, respectively. By contrast, muscone significantly declines the number of TRAP-positive cells in a dose-dependent manner. Thus, muscone suppresses osteoclastogenesis both in BMMs and RAW264.7 cells.

### Muscone Inhibits Actin-Ring Formation and Bone Resorption by Osteoclasts

**Figure 2:**
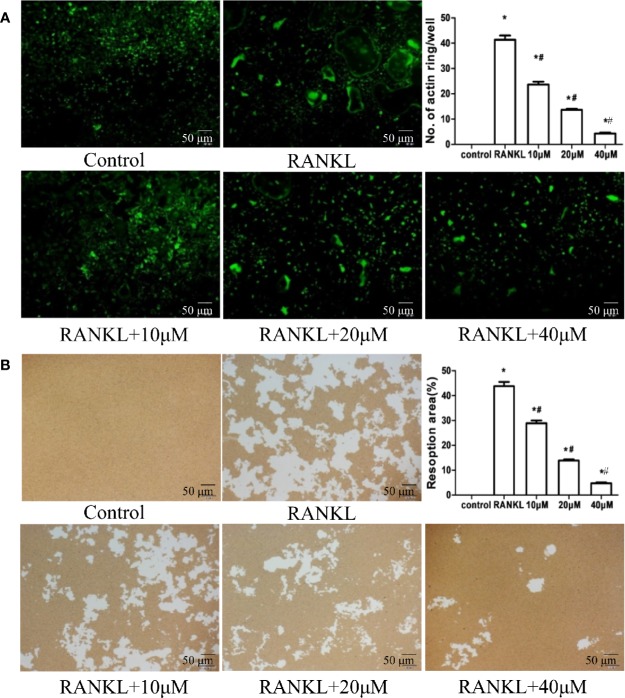
Muscone represses actin-ring formation and bone resorption by osteoclasts. **(A)** Actin-ring formation is observed by FITC-phalloidin staining and quantified. Muscone inhibits the actin-ring formation in a dose-dependent manner. **(B)** Resorption area is quantified by image analysis, and muscone significantly shrinks the resorbed range in a dose-dependent manner. *P < 0.05 vs. the control group, ^#^*P* < 0.05 vs. the RANKL-induced group (original scale bars, 50 μm).

To explore the role of muscone in the osteoclast function and the cytoskeleton formation, actin-ring formation, the most important and apparent process of osteoclastogenesis, is investigated by FITC-phalloidin staining (**Figure 2A**). With the induction of RANKL, BMMs differentiate into mature osteoclasts and forms actin-ring cytoskeleton. However, when cells are incubated with 10, 20, and 40 μM muscone, the size and the number of actin-ring assemblies show a significantly decrease in a dose-dependent manner, which suggests that muscone inhibits the actin-ring formation by mature osteoclasts.

Furthermore, in bone resorption assays, induced with RANKL/M-CSF, RAW264.7 cells form apparent pits on the bone biomimetic synthetic surface, indicating that cells differentiate into mature osteoclasts. However, muscone significantly shrinks the resorbed range, which suggests that muscone can overturn resorption functions of osteoclasts (**Figure 2B**).

### Muscone Has Limited Impact on BMSCs Differentiation

**Figure 3:**
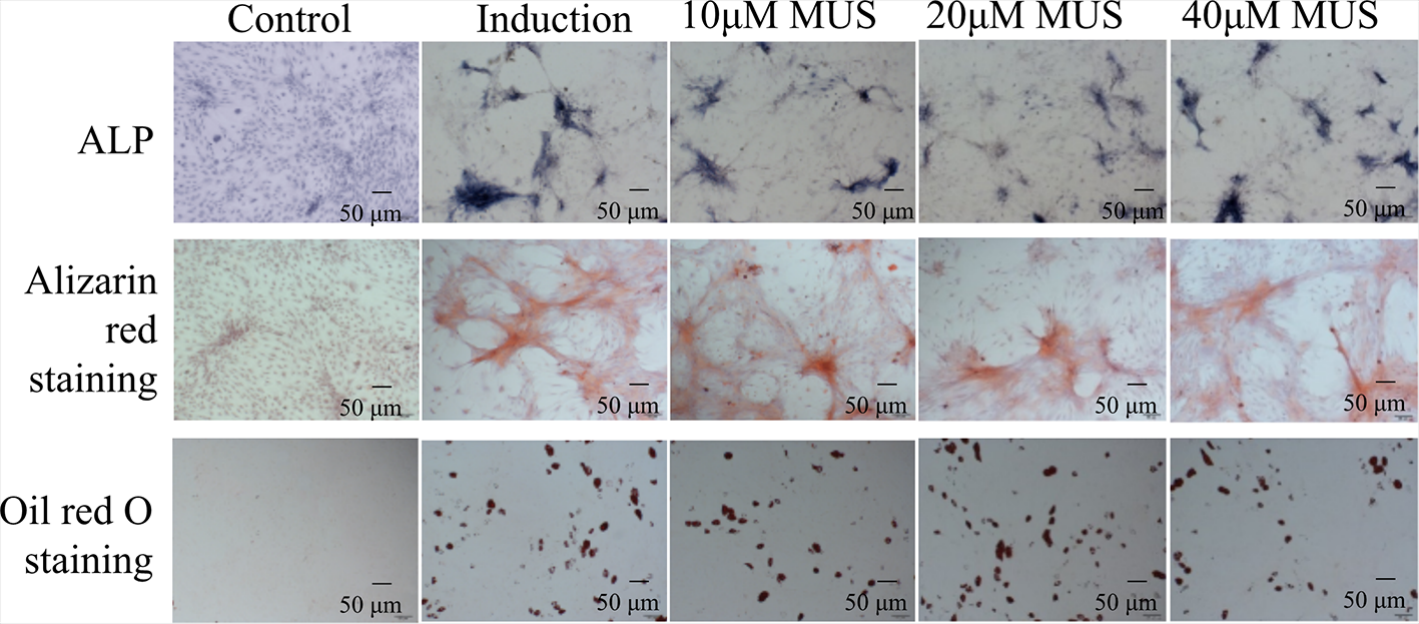
Muscone has limited impact on BMSCs differentiation. Alkaline phosphatase (ALP) staining and alizarin red (ARS) staining show that muscone at 10 μM, 20μM, and 40 μM has little influence on the mineralization of calcium nodules. And the oil red O staining shows that muscone has little consequence on the formation of fat particles *in vitro*.

Besides osteoclasts, osteoblasts differentiated from BMSCs also modulates bone remodeling (Hamam et al., 2014). Therefore, we evaluate the osteogenesis effects of muscone by ALP staining and alizarin red staining. It finds that muscone (40 μM) has little influence on the materialization of calcium nodules (**Figure 3**). In addition, for adipogenesis of BMSCs, the oil red O staining is used, and it shows that muscone has little consequence on the formation of fat particles *in vitro* (**Figure 3**). As a result, muscone presents limited effect on BMSC differentiation and osteogenesis, which indicates that muscone prevents bone loss primarily by affecting osteoclastogenesis.

### Muscone Inhibits Osteoclastogenesis Only at an Early Stage

**Figure 4:**
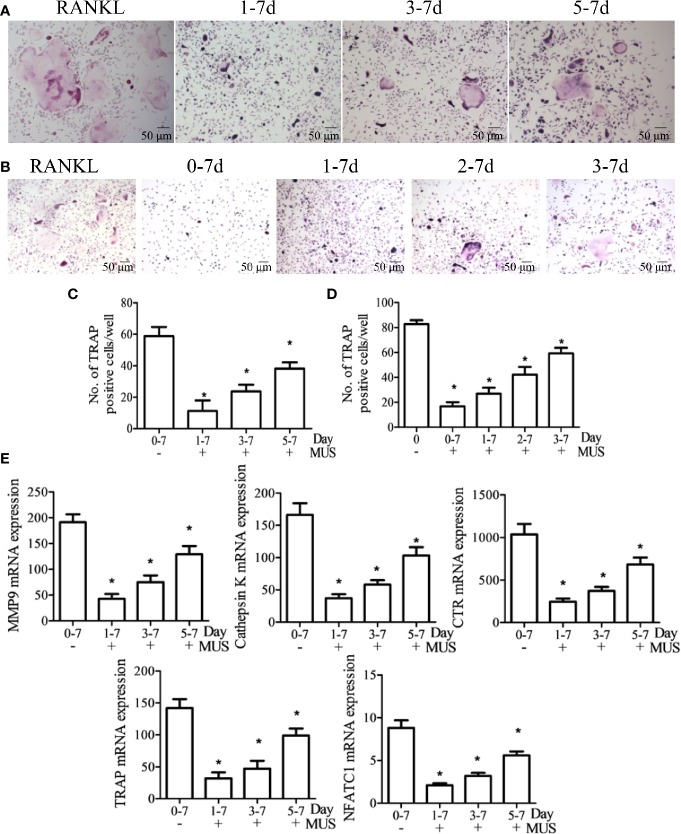
Muscone inhibits osteoclastogenesis only at an early stage. After induction of M-CSF (20 ng/ml) and RANKL (50 ng/ml) on day 0, BMMs and RAW264.7 cells are divided into groups according to the different times that 40 μM muscone is added. **(A, C)** Besides the RANKL group, BMMs are divided into three groups: day 1-7 group, day 3-7 group, and day 5-7 group, since muscone is added on day 1, 3, and 5, respectively. **(B, D)** And similarly, RAW264.7 cells are divided into four groups: day 0-7 group, day 1-7 group, day 2-7 group, and day 3-7 group, as muscone is added on day 0, 1, and 3, respectively. On Day 7 after RANKL stimulation, TRAP staining is performed. **(E)** On day 7, mRNA expression in BMMs, including MMP-9, Cathepsin K, CTR, TRAF6, TRAP, and NFATc1, are detected by RT-PCT for each group. P < 0.05 vs. the control group, #P < 0.05 vs. the RANKL-induced group.

To investigate at which stage muscone inhibits osteoclastogenesis since it is a multistep processes, BMMs and RAW264.7 cells are divided into groups, according to the different days that 40 μM muscone is added after RANKL stimulation (set as day 0). Besides the RANKL group, BMMs are divided into three groups: day 1-7 group, day 3-7 group, and day 5-7 group, since muscone is added on day 1,3, and 5, respectively (**Figures 4A, C**). And similarly, RAW264.7 cells are divided into four groups: day 0-7 group, day 1-7 group, day 2-7 group, and day 3-7 group, as muscone is added on days 0, 1, and 3, respectively (**Figures 4B, D**). On day 7 after RANKL stimulation, TRAP staining is performed since it is supposed to be the granted “mature” day, and downstream mRNA expression including MMP-9, Cathepsin K, CTR, TRAF6, TRAP, and NFATc1 in BMMs are detected by RT-PCR for each group (**Figure 4E**). The results indicate that osteoclast differentiation is remarkably inhibited when muscone is treated on the early stage after RANKL stimulation but is less effective on later stages, suggesting that muscone has primarily effect on the stage of the osteoclastogenesis that monocyte differentiating into osteoclast precursor, but has less effect on mature osteoclasts.

### Muscone Suppresses RANKL-Induced NF-κB Pathways in Osteoclastognesis

**Figure 5:**
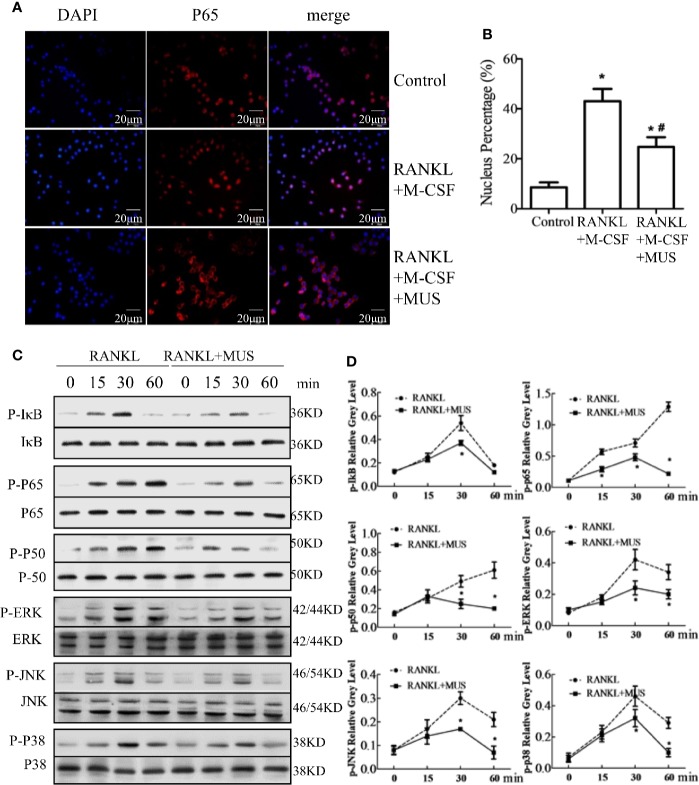
Muscone suppresses RANKL-induced signaling pathways in osteoclastogenesis. **(A, B)** Immunofluorescence staining shows that muscone inhibits RANKL-induced P65 nuclear translocation. **(C, D)** Muscone suppresses phosphorylation of factors in the NF-kB pathway, including P65, P50, and IkB protein. And muscone also blocks phosphorylation of factors in the MAPK pathways in osteoclastogenesis, including ERK, JNK, and P38. *P < 0.05 vs. the control group, #P < 0.05 vs. the RANKL-induced group.

Induced by RANKL, p65 translocates into the nucleus. Immunofluorescence staining shows that RANKL increases p65 location in RAW264.7 cells, but muscone significantly blocks the nuclear translocation of p65 (**Figures 5A, B**). In addition, Western blot results indicate that IκB phosphorylation increases and achieves its peak at 30 min, and the p50 phosphorylation and the p65 phosphorylation intensify at 60 min. Thus, it indicates that the NF-κB pathway is activated after induction of M-CSF and RANKL. However, muscone significantly suppresses phosphorylation levels in RAW264.7 cells (**Figure 5C**). In conclusion, muscone reduces the RANKL-induced NF-κB pathway activation.

### Muscone Suppresses RANKL-Induced MAPK Pathway in Osteoclastogenesis

MAPK pathway is another vital pathway in osteoclastogenesis (Li et al., 2016). For the major subfamilies of the MAPK pathway, such as ERK, p38, and JNK, are examined by western blot. The phosphorylation levels of these factors are analyzed by semi-quantitative detection, and the levels significantly increase after incubating with M-CSF and RANKL, and all reach their peaks at 30 min, but muscone administration inhibits their phosphorylations in RAW264.7 cells (**Figure 5D**). However, quantification results show that levels of these phosphorylation factors are significantly inhibited by muscone (**Figure 5D**). Together, it indicates that the muscone may inhibit osteoclastogenesis *via* targeting the MAPK signaling pathways.

### Muscone Suppresses the RANKL-Induced RANK-TRAF6 Association and Blocks Down-Stream Gene Expression

**Figure 6:**
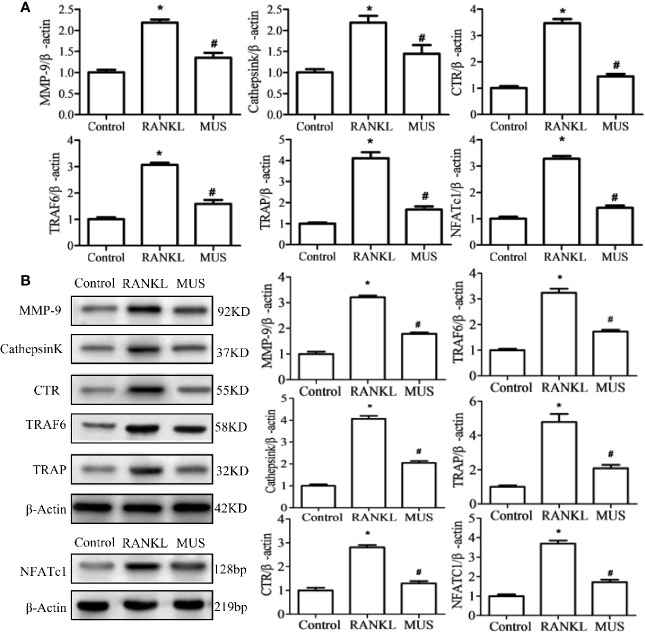
Muscone suppresses the RANKL-induced RANK associated down-stream gene expression. By RT-PCR **(A)** and western blot **(B)**, muscone firstly suppresses the TRAF6 and the over-expressed NFATc1. And then, muscone withdraws expression levels of MMP-9, Cathepsin K, TRAP, and CTR in RAW264.7 cells. *P < 0.05 vs. the control group, #P < 0.05 vs. the RANKL-induced group.

**Figure 7:**
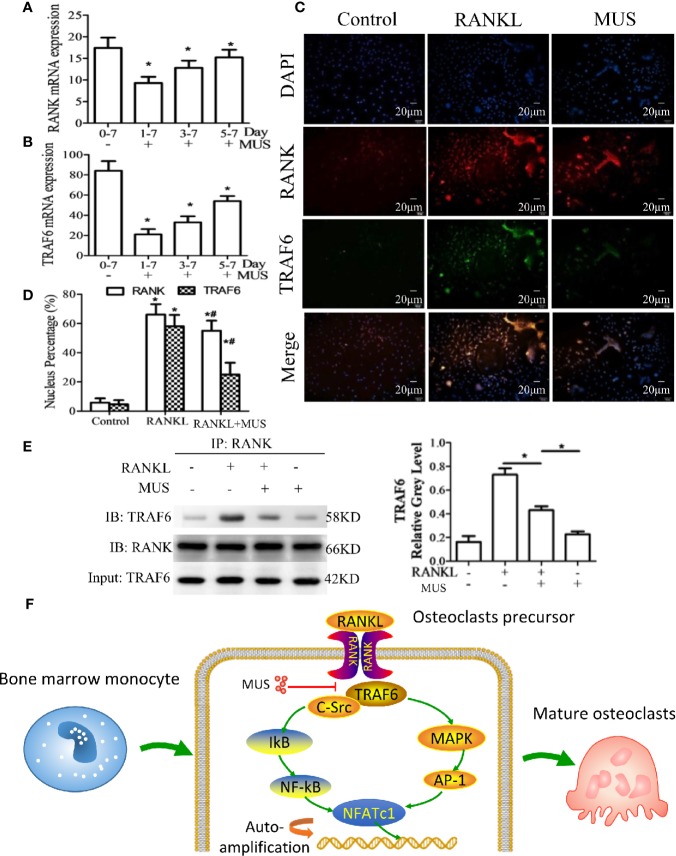
Muscone inhibits RANKL-induced TRAF6 binding to RANK. **(A, B)** After induction of M-CSF (20 ng/ml) and RANKL (50 ng/ml) on day 0, BMMs and RAW264.7 cells are divided into groups according to the different times that 40 μM muscone is added (day 1-7 group, day 3-7 group, and day 5-7 group, since muscone is added on day 1, 3, and 5, respectively). On day 7, RANK and TRAF6 mRNA expression in BMMs are detected by RT-PCR for each group. **(C, D)** RAW264.7 cells are stimulated, with or without RANKL (100 ng/ml) for 20 min, and with or without muscone (40 μM) for 6 h. Cells are then subjected to immunofluorescence staining. The nucleus percentage counting of immunofluorescence staining indicates that muscone significantly downregulated the expression of TRAF6 protein rather than RANK. **(E)** To verify the result, coimmunoprecipitation shows that TRAF6 protein levels of RAW264 cells treated with vehicle or muscone (40 μM) with or without RANKL, measured by Western blot analysis. Input: TRAF6 and IB: RANK was used as loading controls. **(F)** Schematic diagram of the mechanism by which muscone ameliorates RANKL-induced osteoclastogenesis *via* blocking the binding of TRAF6 to RANK. *P < 0.05 vs. the control group, #P < 0.05 vs. the RANKL-induced group.

Since RANKL-induced RANK recruits TRAF6, we examined TRAF6 by RT-PCR and Western blot. It is found that muscone suppresses the expression of TRAF6 significantly after the induction of RANKL (**Figures 6A, B**). In a time-dependent manner described as the above, both of the mRNA levels of RANK and TRAF6 are reduced by muscone at an early stage (**Figures 7A, B**). To further assess whether muscone influences the binding of RANK and TRAF6, immunofluorescence staining (**Figures 7C, D**) and coimmunoprecipitation (**Figure 7E**) are also performed, and as a result, muscone significantly inhibits the binding of TRAF6 to RANK, and suppresses the expression of TRAF6 rather than RANK.

And for down-stream gene expression, NFATc1 is a central regulator in osteoclastogenesis (Wu et al., 2017). In our study, the level of NFATc1 showed by RT-PCR and Western blot in RAW264.7 cells is over-expressed when induced by RANKL. And muscone suppresses its expression. Thus, the expression of osteclastogenesis related genes, such as MMP-9, Cathepsin K, TRAP, and CTR, are significantly down-regulated added with muscone (**Figure 6**).

### Muscone Prevents Bone Loss in OVX Mice by Suppressing Osteoclastogenesis

**Figure 8:**
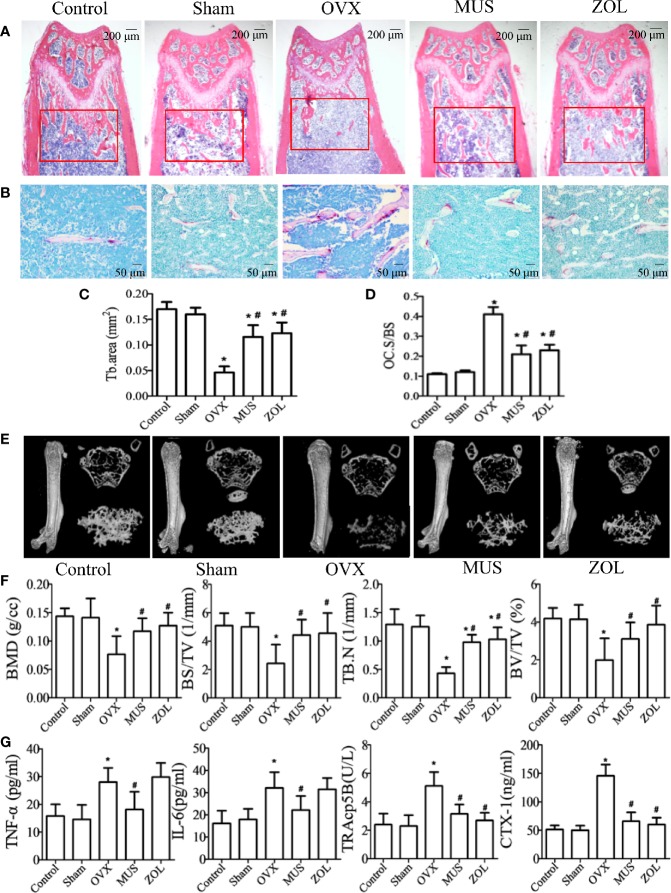
Muscone prevents bone loss in OVX mice by suppressing osteoclastogenesis. **(A, C)** Mice are sacrificed 6 weeks after operation, and H&E staining of femoral shows that the OVX group has a significant trabecular bone loss, and the muscone group has more trabecular area. **(B, D)** TRAP staining shows that muscone significantly depresses osteoclasts differentiation. **(E)** For each femur, 100 section planes from the growth plate are scanned, and bone structure images in two-dimensional and three-dimensional version are reconstructed. **(F)** Bone mineral density (BMD), bone volume/total volume (BV/TV), bone surface area/total volume (BS/TV), and trabecular number (Tb.N), within the selected metaphyseal region, are showed in the charts. **(G)** TNF-a, IL-6, TRAcp5B, and CTX-1 are examined in serum. *P < 0.05 vs. the control group, ^#^*P* < 0.05 vs. the OVX group.

In postmenopausal osteoporosis, as the level of estrogen decreases, the genesis and activity of osteoclasts are enhanced. And it leads to an imbalance in bone hemostasis and inflammatory response (Sapir-Koren and Livshits, 2017; Chen et al., 2018). Therefore, we examined the effects of muscone on OVX mice. Mice are sacrificed 6 weeks after operation. Hematoxylin and eosin (H&E) staining and micro computed tomography (CT) show that the OVX group has a significant trabecular bone loss, and the muscone group has more trabecular area (**Figures 8A, E**). In addition, detailed results for HE staining and micro CT analysis are shown in **Figures 8B, F**. Furthermore, the number of TRAP staining positive osteoclasts significantly increases in the OVX group, and muscone significantly decreases the number of osteoclasts (**Figures 8B, D**).

For pro-inflammatory cytokines, we examined the serum levels of tumor necrosis factor (TNF)-α, and interleukin (IL)-6. And for osteoclastogenesis serum markers, we inspect C-telopeptide of type I collagen (CTX-1) and tartrate-resistant acid phosphatase (TRAcp5B). Compared with the control group, levels of the above serum factors are significantly higher in OVX group. However, treated with muscone, the serum levels significantly decrease. As a result, muscone suppresses pro-inflammatory cytokines secretion and osteoclast activity in OVX mice (**Figure 8G**).

## Discussion

In this study, we demonstrated that muscone suppressed osteoclastogenesis *in vitro* and reversed OVX-induced bone loss *in vivo*. To our knowledge, it is the first report that muscone can ameliorate bone-related pathology. For further molecular mechanisms, we found that muscone inhibited the binding of TRAF6 to RANK induced by RANKL, thus blocking NF-kB and MAPK pathways, and down-regulating related gene expression.

Bone homeostasis is mediated through continuous formation by osteoblasts and resorption by osteoclasts. Over-activated osteoclasts can induce a number of pathologic and osteopenic diseases, such as postmenopausal osteoporosis, rheumatoid arthritis, lytic bone metastasis, and Paget disease (Wakchoure et al., 2009; Rana et al., 2018). After menopause, estrogen deficiency leads to the higher levels of RANKL and pro-inflammatory factors, including IL-6 and TNF-α (Sun et al., 2018). The over-activation of RANKL signaling pathways recruits the RANK/TRAF6 association and thus promotes the reproduction of osteoclasts (Chen et al., 2018; Lee et al., 2018). As a result, it is a potential therapeutic target for PMOP *via* inhibiting osteoclastogenesis.

Natural products are believed very helpful in traditional medicine, and their bioactive function and mechanism have been largely explored these years. Muscone was identified in the natural extract of deer musk as early as 1906. It is used in a large number of fine perfumes (Callejo et al., 2016). And it is also known as an effective drug with anti-inflammatory effects for multiple systems (Wang et al., 2014; Liu et al., 2014; Du et al., 2018). Since postmenopausal osteoporosis is regarded to be deteriorated by the inflammatory status due to the withdrawal of estrogen, and osteoclasts are then induced by pro-inflammatory cytokines, we hypothesized that muscone might also have effects on osteoclastogenesis and bone loss.

To exclude the cytotoxic effects of muscone, we firstly performed the MTT analysis. And then *in vitro* study, muscone was proved to suppress the formation of mature osteoclast. However, it showed to inhibit the osteoclastogenesis at an early stage. Since M-CSF induces the proliferation of osteoclast precursor cells, and RANKL induces subsequent differentiation into mature osteoclasts (Song et al., 2018), our results showed that muscone might block the differentiation at the former step.

In addition, RANKL induces its receptor RANK, and recruits TRAF6 to form RANK/TRAF6 association (Liu et al., 2018). It is reported that a block on the single TRAF6-binding site is sufficient for osteoclastogenesis, since TRAF6-binding motif, when interacts with TRAF6, can lead to the final induction of NFATc1 (Gohda et al., 2005; Hu et al., 2019). In this study, muscone could reduce both levels of RANK and TRAF6, which showed less change on RANK when compared with TRAF6. Since the binding of TRAF6 to RANK was lessened when treated by muscone in both results of immunofluorescence staining and coimmunoprecipitation, downstream pathways were blocked, including NF-κB and MAPK pathways and then inflammatory response. Consequently, the levels of TNF-α and IL-6 were reduced *in vivo*. Furthermore, the reduced inflammatory factors might then lead to the decrease of the expression of RANK (Zaiss et al., 2019). In addition, as a small molecular, muscone might be easy to penetrate the cell membrane to inhibit the RANK-TRAF6 interaction. As a result, it suggested that muscone might competitively bind to the TRAF6-binding sites and then inhibit osteoclastogenesis.

Furthermore, the downstream signaling pathways were then inhibited (Wu et al., 2012; Wang et al., 2016). NF-kB pathway is essential for osteoclastogenesis, and it has been proven by numerous genetic and pharmacological studies (Teitelbaum and Ross, 2003). In our study, muscone suppressed the expression of NF-kb pathway induced by RANKL, including phosphorylation of IkBa, p65, and p50, and nuclear translocation of p65. Furthermore, MAPK pathway is also well known and activated by RANKL induction (Choe et al., 2015). ERK, JNK, and p38 were phosphorylated in this study, and inhibited by muscone. For further cascade reaction, NFATc1 presents as a central regulator in osteoclastogenesis that facilitates the related down-stream genes (Kim et al., 2018; Chen et al., 2018). In our studies, muscone reversed the over-expression of NFATc1 in RAW264.7 cells. And then, the expression levels of following related markers, including MMP-9, Cathepsin K, TRAP, and CTR, were significantly down-regulated.

However, this study has several limitations. On one hand, although we deduced that muscone mainly affected osteoclastogenesis with results that BMSCs osteogenesis and adipogenesis were not promoted by muscone, its effects on osteogenesis remained a question. On the other hand, the effects of muscone on PMOP patients need to be clarified.

## Conclusion

Our research demonstrated that muscone might ameliorate OVX-induced bone loss in mice and osteoclast differentiation *in vitro*, by blocking the TRAF6-mediated association to RANK induced by RANKL, and then suppressing NF-κB and MAPK signaling pathways and down-regulating related gene expression.

## Data Availability Statement

All datasets generated for this study are included in the article/**Supplementary Material**.

## Ethics Statement

The animal study was reviewed and approved by the ethical committee of Shanghai Changhai Hospital.

## Author Contributions

XZha and ML designed this study. XZha and ZY finished the paper draft. KC, YY and XZho conducted the methodology. MC, CHe, and CHu did the analysis. MY, XZho, and BL finished data calculation. KC, ZY, and XW did the staining and visualization. YZ, YB and ML did the supervision. All authors contributed to manuscript preparation.

## Funding

This study was supported by National Natural Science Foundation of China (81701199); Pilot Project Clinical Collaboration of Traditional Chinese Medicine and Western Medicine for Major and Difficult Diseases (ZY[2018-2020]-FWTX-2005); Shanghai Science and Technology Commission Fund (17441900500, 16DZ0504000); Foundation of Changhai Hospital (CH201717).

## Conflict of Interest

The authors declare that the research was conducted in the absence of any commercial or financial relationships that could be construed as a potential conflict of interest.

## Abbreviations

PMOP, postmenopausal osteoporosis; M-CSF, macrophage colony-stimulating factor; RANKL, receptor activator for NF-kB ligand; BMMs, bone marrow monocytes; TRAF, TNF receptor–associated factor; NF-kB, nuclear factor-kB; MAPK, mitogen-activated protein kinase; NFATc1, nuclear factor of activated T-cell cytoplasmic 1; MMP-9, matrix metalloproteinase 9; CTR, calcitonin receptor; TRAP, tartrate-resistant acid phosphatase; OVX, ovariectomy; FBS, fetal bovine serum; CT, computed tomography; BMD, bone mineral density; BS/TV, bone surface area/total value; BV/TV, bone value/total value; Tb.N, Trabecular number; EMSA, electrophoretic mobility shift assay; H&E, hematoxylin and eosin; TNF, tumor necrosis factor; IL, interleukin; CTX-1, C-telopeptide of type I collagen; TRAP, tartrate-resistant acid phosphatase; MUS, muscone; ALP, Alkaline phosphatase; ARS, alizarin red; ZOL, zoledronic acid.
