# Supplementary material for: Muscone Ameliorates Ovariectomy-Induced Bone Loss and Receptor Activator of Nuclear Factor-κb Ligand-Induced Osteoclastogenesis by Suppressing TNF Receptor–Associated Factor 6-Mediated Signaling Pathways
Source: Front Pharmacol. 2020 Mar 20;11:348. doi: 10.3389/fphar.2020.00348 (PMC7099619; doi:10.3389/fphar.2020.00348)
Supplement: Supplementary file 1 [file Table_1.docx]

**Supplemental Table S1.** PCR primers of downstream biomarkers.

| **Primer** | **Sequence（5'-3'）** |
| --- | --- |
| *CTR*(mouse)-RT-F | AAGGAGGTCCAGAGTGAAAAGG |
| *CTR*(mouse)-RT-F | CGGAGTCAGTGAGATTGGTAGG |
| *MMP*-9(mouse)-RT-F | GAATAAAGACGACATAGACGGC |
| *MMP*-9(mouse)-RT-R | ATAGTGGGACACATAGTGGGAG |
| *TRAP*(mouse)-RT-F | CATTTTCGGGGTGTTTCTCTAT |
| *TRAP*(mouse)-RT-R | TCTCTTTACCATCCTCCTGTGT |
| *TRAF6*(mouse)-RT-F | CCACAATCCCACGGAACCCCAA |
| *TRAF6*(mouse)-RT-R | CGAAGGCCACCCATGTCAAAGC |
| *NFATC1*(mouse)-RT-F | TCGGCGGGAAGAAGATGGTGCT |
| *NFATC1*(mouse)-RT-R | CCTCTGGTTGCGGAAAGGTGGT |
| *CathepsinK*(mouse)-RT-F | CCAGAAGCAGTATAACAGCAAG |
| *CathepsinK*(mouse)-RT-F | TATGTATGGACACCAAGAGAGG |
| β-actin(mouse)-RT-F | GTCCCTCACCCTCCCAAAAG |
| β-actin(mouse)-RT-F | GCTGCCTCAACACCTCAACCC |
